# Identification of Methamphetamine Abstainers by Resting-State Functional Magnetic Resonance Imaging

**DOI:** 10.3389/fpsyg.2021.717519

**Published:** 2021-08-30

**Authors:** Tingting Dong, Qiuping Huang, Shucai Huang, Jiang Xin, Qiaolan Jia, Yang Gao, Hongxian Shen, Yan Tang, Hao Zhang

**Affiliations:** ^1^School of Computer Science and Engineering, Central South University, Changsha, China; ^2^National Clinical Research Center for Mental Disorders, and Department of Psychiatry, The Second Xiangya Hospital of Central South University, Changsha, China; ^3^Hunan Key Laboratory of Psychiatry and Mental Health, Hunan Medical Center for Mental Health, Institute of Mental Health of Central South University, Chinese National Technology Institute on Mental Disorders, Changsha, China; ^4^The Fourth People’s Hospital of Wuhu, Wuhu, China

**Keywords:** Methamphetamine, classification, independent component analysis, sliding window, brain network, K-means clustering

## Abstract

Methamphetamine (MA) can cause brain structural and functional impairment, but there are few studies on whether this difference will sustain on MA abstainers. The purpose of this study is to investigate the correlation of brain networks in MA abstainers. In this study, 47 people detoxified for at least 14 months and 44 normal people took a resting-state functional magnetic resonance imaging (RS-fMRI) scan. A dynamic (i.e., time-varying) functional connectivity (FC) is obtained by applying sliding windows in the time courses on the independent components (ICs). The windowed correlation data for each IC were then clustered by k-means. The number of subjects in each cluster was used as a new feature for individual identification. The results show that the classifier achieved satisfactory performance (82.3% accuracy, 77.7% specificity, and 85.7% sensitivity). We find that there are significant differences in the brain networks of MA abstainers and normal people in the time domain, but the spatial differences are not obvious. Most of the altered functional connections (time-varying) are identified to be located at dorsal default mode network. These results have shown that changes in the correlation of the time domain may play an important role in identifying MA abstainers. Therefore, our findings provide valuable insights in the identification of MA and elucidate the pathological mechanism of MA from a resting-state functional integration point of view.

## Introduction

Methamphetamine (MA) is synthesized on the basis of ephedrine, which has the effect of mental stimulation (Chen et al., 2021). MA is one of the most widely abused illegal substances in the world, but unfortunately, there are still very few studies on detoxification after a period of time (Huang et al., 2021; Li et al., 2021). Due to the high addiction, it is prohibited in many countries. The abuse and addiction of MA has become one of the difficult problems threatening world health (Manzanares et al., 2018; Nicolas et al., 2021), which not only harms the health of addicts, but also hinders the economic development.

Methamphetamine can cause brain structure and functional impairments, whether the impairment still exists after a recovery period is not yet known. To study this problem, we collected neuroimaging data from abstaining MA-independent individuals (for at least 14 months). A better understanding of functional connectivity in the brains of MA abstainers will help to explain abnormal behavioral syndromes and to perform objective diagnosis of MA abstainers. As an effective method to analyze brain pathological patterns, resting-state functional magnetic resonance imaging (RS-fMRI) can reflect the spontaneous brain activity in humans (Rashid et al., 2016; Salman et al., 2018; Stoehr et al., 2021). However, RS-fMRI generates high dimension data. The key challenge to analyze RS-fMRI data is how to extract effective features. In the past, most studies of MA mainly focused on region of interest (ROI), which usually required prior knowledge to choose brain region in the network (Taheri et al., 2016). However, mounting evidences indicate that neurodegenerative processes are associated with the alterations in functional connectivity across the whole brain (Tang et al., 2018). There are few studies on the abnormal functional connection patterns of MA abstainers in the whole brain. One work on this topic showed that compared with healthy controls, MAs showed low network function in the cerebellum and high intra-network function in the post-significant network, proving that there is indeed a change in the brain function network(Jiang et al., 2021).

To analyze the high dimensional RS-fMRI data, it is possible to use kernel principal component analysis to identify the attention-deficit hyperactivity disorder though the accurate rate was less than 80% possibly due to the noisy data (Salimi-Khorshidi et al., 2014; Abrol et al., 2019). Typical independent component analysis (ICA) is a data-driven method (Allen et al., 2014; Qiu et al., 2019), which can eliminate the artificial error and extract the largest spatial independent component (Glasser et al., 2018). It can be used to obtain the brain network while removing noise and separate different but overlapping activities (Zhang et al., 2019). When ICA is used to find hidden sources from a group of observation or measurement data, each source has the greatest independence. ICA is a combination of spatial components, and each component is related to time courses (TCs) (Calhoun et al., 2005; Xie et al., 2017).

Some more recent methods for RS-fMRI data classification use functional connection as the input feature (Rashid et al., 2016). Although some progress has been made, the dynamics of time courses are ignored. In diagnosing and distinguishing complex mental diseases such as schizophrenia and Alzheimer’s disease, the overlooked time information is likely to become a key point in disease analysis (Yan et al., 2019). Sliding window correlation is a popular method used by most dynamic FC studies to capture the dynamics in TCs (Keilholz et al., 2013; Thompson et al., 2013; Wilson et al., 2015; Shakil et al., 2016; Vakamudi et al., 2020). When drawing a window on TCs, the two edges of the window will move during the acquisition process. It can better capture the dynamic changes of brain activity, thus enhancing the understanding of normal cognition and changes caused by brain diseases (Abrol et al., 2017).

After the completion of sliding window correlation, clustering is usually used to find the number of states and time points in the scanning process (Damaraju et al., 2014; Shakil et al., 2014). In the past few decades, various clustering algorithms have been proposed. Among these algorithms, k-means is a popular choice because it is simple, efficient, and has moderate but stable performance on different problems (Himberg et al., 2004). Since each person has multiple sliding windows, here we take the number of windows for every person in each class as the new features for classification. The decision tree is used as the classifier (Douglas et al., 2011), where the subject searches along a single path from the root to the leaf, and the path depends on the characteristics of the sample (Hehn et al., 2019). The decision tree can handle uneven data without standardizing and quantifying the data, and the logic is simple and intuitive.

In this paper, we exploit ICA, sliding window correlation, k-means clustering and decision tree to perform the data analysis. The motivation is to reduce the dimensionality of high-dimensional brain data for subsequent analysis. We propose the feature filtering method with multiple classification criteria, and the experimental results have shown its effectiveness in rendering the differences between the normal people and MA abstainers in their brain networks.

## Materials and Methods

### Ethics Statement

All subjects were fully informed of the nature of the study and all gave their written consent regarding participation. This study was approved by the local ethical committee of the Second Xiangya Hospital of Central South University Institutional Review Board for clinical research.

### The Overall Introduction of the Method

**FIGURE 1:**
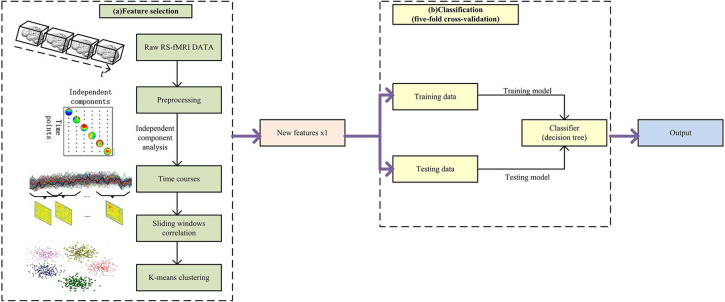
The framework of the proposed method.

In order to discriminate the MA abstainers from the normal people, we developed a data-driven classifier that incorporates five steps: preprocessing, sliding windows, k-means clustering and classification. Figure 1 shows the overall flowchart.

### Data

We selected a data set containing 91 individuals (44 normal individuals, 47 MA abstainers), which was provided by the Second Xiangya Hospital of Central South University Medical Academy. The data collection was completed on the 3.0T MRI scanning system produced by Siemens. Everyone was required to wear sponge earplugs and noise-canceling headphones to lower noise, and elastic sponges were also used to fix the head to reduce head movement. When scanning in compartments, the even-numbered layer is scanned first, and then the odd-numbered layer. Each individual was collected 225 contiguous whole-brain resting-state functional images with the following parameters: number of slices = 36, TR = 2,000 ms, TE = 30 ms, FOV = 220 mm, flip angle = 80 degrees, slice thickness = 4 mm, image dimension = 64 × 64 × 36, and voxel size = 3.4 mm × 3.4 mm × 4 mm.

### Data Preprocessing

In the preprocessing, the SPM^1^ and DPASF (Yan et al., 2016) toolboxes were used. Due to the magnetic saturation, we removed the first ten volumes for each person. Then we proofread the scanning time point of each layer image, and the head motion correction was used to exclude subjects with excessive head movement. Next, we used the Echo-Planer Imaging (EPI) template of the Montreal Neurologic Institute (MNI) space to standardize the image registration to 3 mm × 3 mm × 3 mm (Brandman et al., 2021; Fan et al., 2021; Peng et al., 2021), and finally the Gaussian kernel with a half-height full-width value of 6 mm was used for spatial smoothing to reduce spatial noise (Maniar et al., 2021; van Buuren et al., 2021).

For quality control, participants with head movements greater than 2 mm or rotation parameters greater than 2 degrees were excluded. Forty one normal individuals and 40 MA abstainers were retained.

### Independent Component Analysis

**FIGURE 2:**
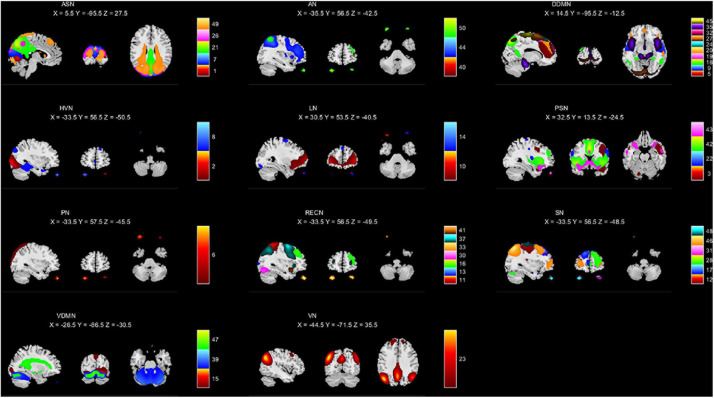
Intrinsic connectivity networks (a total of 44) arranged into groups: ASN, AN, DDMN, HVN, LN, PSN, PN, RECN, SN, VDMN, and VN. The associated number in each group indicates the number of ICNs included the group.

We used the GIFT toolkit^2^ to extract independent components from the pre-processed data. After estimating the optimal numbers of independent components using the MDL criterion (Li et al., 2007), we selected 50 independent components. The independent components estimated from the GIFT were processed by initializing parameters, group data reduction, calculating ICA, back reconstructing, calibrating components, and group stats. Then we used the resting state network (RSN) (Du and Fan, 2013; Du et al., 2017) template to generate the mask of 11 networks shown in Figure 2, including anterior salience network (ASN), auditory network (AN), dorsal default mode network (DDMN), higher visual network (HVN), language network (LN), posterior salience network (PSN), precuneus network (PN), right executive control network (RECN), sensorimotor network (SN), ventral default mode network (VDMN) and visuospatial network (VN). We calculated the correlation coefficients between the independent components and each mask, and allocated the independent components to the brain network with the highest correlation. The calculated correlation coefficients were placed in the attachments. Figure 2 shows the independent components in each brain network, and the area of each independent component is marked with a different color.

### Sliding Windows Correlation

After going through the above steps, each participant has 44 time courses. Every time course has 215 time points. We used the sliding windows on the time axis. The window size between 30 and 60 s (15-30TR) leads to a relatively small impact on the dynamics, and can help correctly identify the cognitive states (Zalesky and Breakspear, 2015). Thus, the size of a sliding window was chosen as 25 TR with step size chosen as two. Here each participant is measured by (215−25)/2 = 95 sliding windows. For each sliding window, the correlations in pairs of time courses were calculated. Thus 44 × 44 symmetric matrices would be generated for each sliding window. Next, the upper triangular matrix of this symmetric matrix was extracted and converted it into a row vector. Then a 95 × 946 (946 = 44 × 43/2) dimensional sliding window feature matrix was produced for each participant, and each row represents the correlation of the independent components at that time point.

### Feature Selection

The feature matrices were calculated with the k-means clustering algorithm. The optimal number of clusters was determined to be 5 using the elbow criterion (Plis et al., 2018). We clustered the sliding window feature matrices of all participants by row. For everyone, we counted the number of windows gathered in each category, and these numbers were used as new features. After that, a new matrix of 1 × 5 dimensions was generated for each participant, which is the input to the decision tree for classification. Fivefold cross-validation was used and the metrics of the five times were averaged as the total results. The feature matrices of all participants were divided into five parts. Four parts were selected as the training set and the remaining part was used as the test set.

Different indicators are used to measure the method, including sensitivity, specificity, precision, accuracy, and F1 score. Sensitivity is the proportion of the true normal people who are correctly identified. Specificity is the proportion of true MA abstainers who are correctly identified. Accuracy is the percentage of people who are completely matched. Precision is the proportion of predicted normal people who are correctly identified. F1 score is the harmonic average of precision and sensitivity. The specific calculation methods are shown in below.

(1) $$\text{Sensitivity}=\frac{T\,P}{T\,P+F\,N}$$

(2) $$\text{Specificity}=\frac{T\,N}{T\,N+F\,P}$$

(3) $$\text{Precision}=\frac{T\,P}{T\,P+F\,P}$$

(4) $$\text{Accuracy}=\frac{T\,P\,T\,N}{T\,P+T\,N+F\,P+F\,N}$$

(5) $$F_{1}\,\mathrm{score}=\frac{2*P\,r\,e\,c\,i\,s\,i\,o\,n*\text{Sensitivity}}{P\,r\,e\,c\,i\,s\,i\,o\,n+\text{Sensitivity}}$$

Here TP denotes the number of true positives (number of the normal people who are correctly identified) and TN is the number of true negatives (number of the MA abstainers who are correctly identified). FP denotes the number of false positives (number of the MA abstainers who are classified as the normal people), while FN is the number of false negatives (number of the normal people who were classified as the MA abstainers).

## Results

**FIGURE 3:**
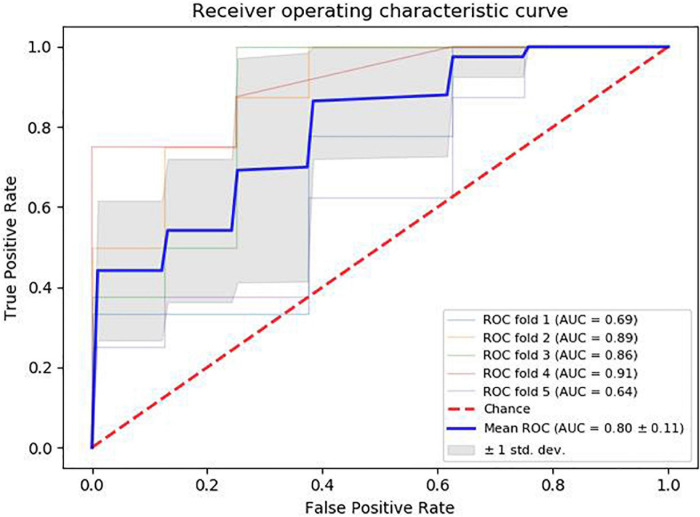
ROC curves and AUC values are shown in the picture.

Various indicators of the model are calculated (accuracy: 82.3%, precision: 77.7%, specificity: 77.7%, sensitivity: 85.7, F1 score: 81.5%). The ROC curve is shown in Figure 3.

In order to exclude the influence of head movement and age, we ensured that there is no significant difference between the parameters of MA abstainers and normal people during preprocessing. The head movement parameters of the remaining subjects are shown in Table 1. The *p*-value is obtained by the two-sample *t*-tests, and the data in the table is represented by the mean ± standard deviation.

**TABLE 1:** Head movements and age information of the subjects.

	**MA abstainers**	**Normal people**	***P* value**
Age	32.97 ± 6.85	34.94 ± 6.9	0.2118
Head motion (MEAN_FD_ JENKINSON)	0.0655 ± 0.03539	0.06832 ± 0.0443	0.7583

**FIGURE 4:**
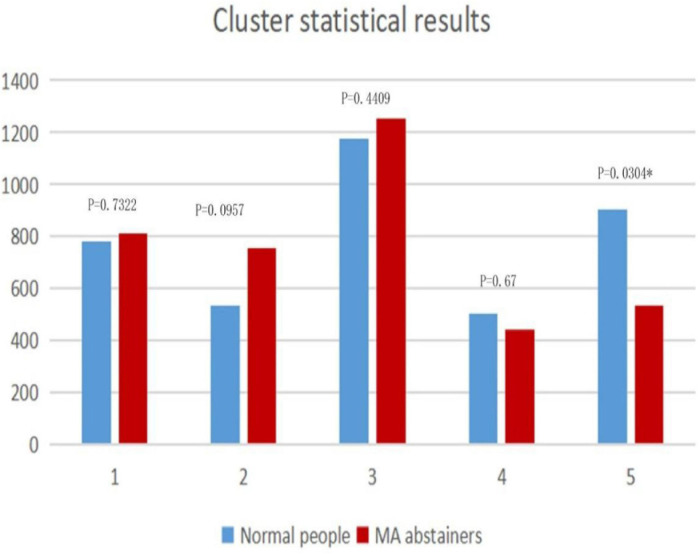
The figure shows the distributions in clusters. The horizontal axis represents the state of each cluster, and the vertical axis is the total number of windows in each category. The *p*-value of the two-sample *t*-test for each state is marked on the histogram.

We can see the feature distributions of various states in Figure 4. In the fifth state, there is a significant difference (*p* < 0.05) in the number of windows between normal people and MA abstainers. In the remaining states, there were no significant differences between normal people and MA abstainers.

**FIGURE 5:**
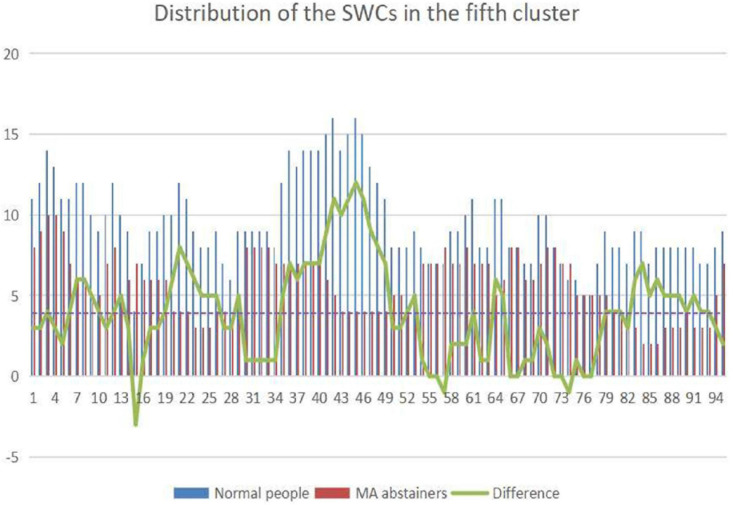
The picture shows the distribution of the sliding windows gathered in the fifth state of normal people and MA abstainers. The abscissa represents the label of the sliding windows, and the ordinate represents the number of sliding windows. The blue column represents the total number of normal people classified into a certain category, and the red one is MA abstainers. The green broken line indicates the difference in the total number of sliding windows.

The number of windows which is clustered to the fifth state in every sliding window is shown in Figure 5. The sliding windows that are higher than the mean of the difference are selected. The sliding windows of normal people and MA abstainers are selected for two-sample *t*-test and FDR multiple ratio correction, where the correlations with *p* value less than 1e-4 (Fayers, 2008) are selected for further analysis.

**FIGURE 6:**
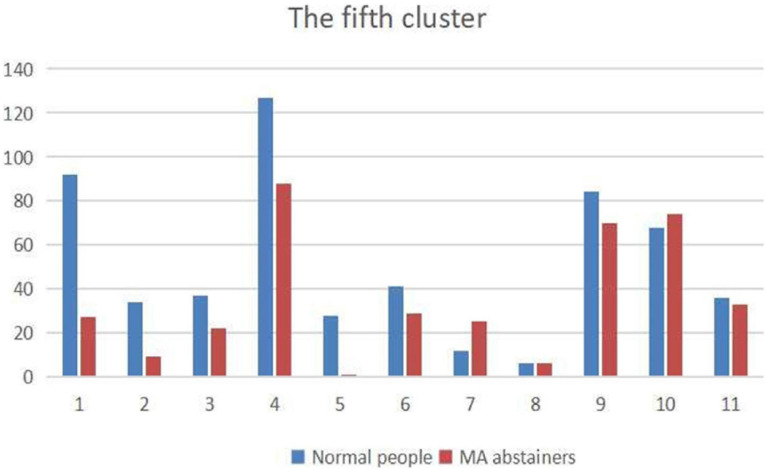
The figure shows the distribution of independent components belonging to the brain networks. The names of the brain networks labeled 1–11 are: ASN, HVN, PSN, DDMN, PN, VDMN, LN, VN, RECN, SN, and AN.

Since the values in the sliding windows reflect the correlation between two independent components in the time courses, and each independent component belongs to a brain network. We draw a distribution map of the brain network of independent components in the Figure 6, and the largest proportion is DDMN.

**FIGURE 7:**
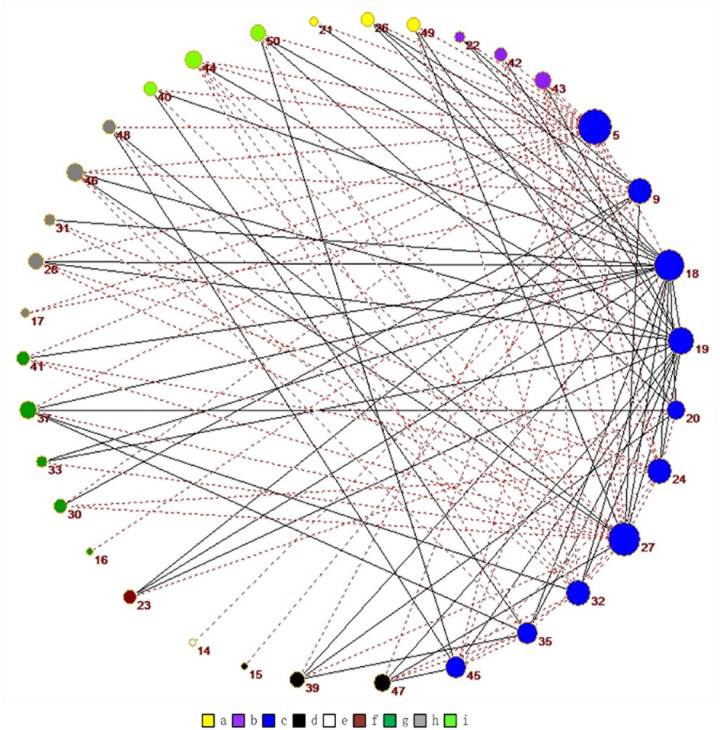
The graph shows all the correlations that are significantly different for normal people and MA abstainers in the DDMN. Among them, the red dotted line indicates the higher correlation of normal people while the gray one means the higher correlation of MA abstainers. The colors are related to different brain networks. The brain networks of a-i in the figure are ASN, PSN, DDMN, VDMN, LN, VN, RECN, SN, and AN.

**FIGURE 8:**
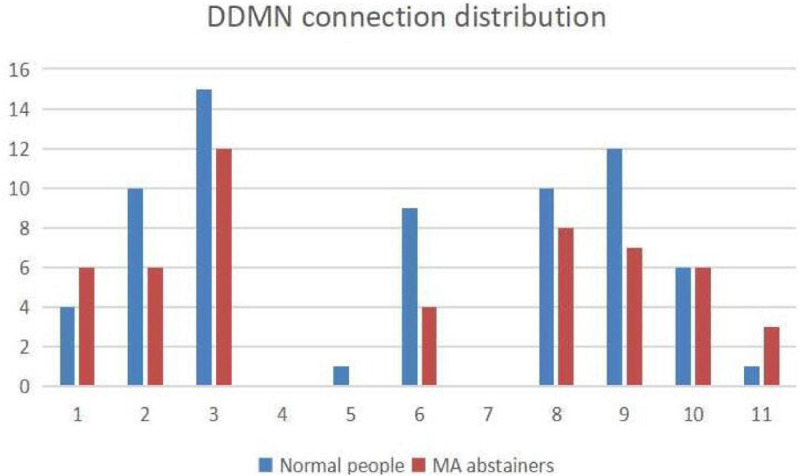
The figure shows the distribution of connection with each brain network in DDMN. The numbers 1–11 represent the corresponding brain networks: ASN, HVN, PSN, DDMN, PN, VDMN, LN, VN, RECN, SN and AN. The blue histogram indicates the number of normal people’s connections in this brain network, and the red histogram indicates the number of MA abstainers’ connections in this brain network.

As shown in Figure 7, we use Pajek^3^ to draw the relationship among the independent components in the DDMN. In Figure 8, in addition to ASN and AN, the correlation of the normal people between DDMN and other brain is higher. That is to say, the connection between DDMN and ASN, DDMN, and AN in MA abstainers is more intimate, but this degree of intimacy is not highlighted in normal people. However, the correlation between DDMN and itself and LN is same between MA abstainers and normal people.

In the time domain, the difference in DDMN between normal people and MA abstainers is the largest, and the results of the two-sample *t*-test in the spatial domain are listed in Table 2. The data shown in the table indicates that the difference in the time domain does not affect the spatial domain.

**TABLE 2:** The *p*-values of the components are shown in the table.

**The name of brain networks**	***p*-values of *t*-test**
ASN	0.4809
AN	0.4481
DDMN	0.4889
HVN	0.506
LN	0.4889
PSN	0.4896
PN	0.5569
RECN	0.5046
SN	0.495
VDMN	0.4814
VN	0.5051

## Discussion

This study shows that MA abstainers can be distinguished from healthy controls by whole-brain RS-fMRI with high resolution. In addition, we find that the abnormal correlations on DDMN with other brain networks, indicating that MA may cause changes in the correlation of these brain networks in the time domain, but these differences are not synchronized in the space domain. The results of this study show that even after long-term withdrawal of MA, certain brain regions are exposed of drug related clues.

Previous research demonstrates that the abuse of MA has serious damage to various organs of the human body, including the brain. DDMN can be regarded as the background network of the brain (Yuan et al., 2021). The main feature is that its functional activity is higher in the resting state than in the motion state, which is related to memory and information processing. We found that DDMN plays the most important role in distinguishing normal people from MA abstainers, which is consistent with the results of many mental diseases. It shows abnormal functional connections in the MA abstainers, agreeing with the previous research results (Peng et al., 2021). This indicates that DDMN may be involved in the pathogenesis of mental diseases, and the results are related to the selected statistical methods, data analysis methods, sample size, selection of psychotropic drugs during treatment and other factors (Luo et al., 2019).

Emerging evidences suggested that DDMN plays a key role in processing consciousness, self-reflection, and episodic memory (Higgins et al., 2021). For people who are addicted to MA, they are weak in self-control and generally require external compulsory drugs to assist with withdrawal. These evidences coincide with our results that the brain network of MA abstainers after withdrawal for a period of time still has a big difference in DDMN. Long-term use of MA can lead to dysfunction of the brain network, and similar experiments have also found that the brain networks of MA abstainers have been damaged (Su et al., 2020). We also found that similar damage still exists after a period of treatment. Generally speaking, the correlation between DDMN and other brain networks is higher for normal people.

Dorsal default mode network plays the role of a central hub in prediction, which can reduce classification errors. In research on substance use disorders, it was found that DDMN is related to impaired self-awareness. This impairment manifests itself as the inability to convert internal or external stimuli to the individual into feedback to the self. The impairment of self-awareness also promotes uncontrolled drug use and also decreased sensitivity to the effects of drugs (Zhang et al., 2019). The ASN of MA abstainers is less correlated with other brain networks. After using MA, it will cause high-frequency auditory hallucinations (Liu et al., 2020).

After a period of detoxification, MA abstainers and normal people still have differences in some brain networks. The results of successful classification showed that the correlation between the independent components of MA abstainers and the control group was still significantly different in some brain networks. Drug addiction is a chronic and recurrent disease, which causes a huge global health burden. At present, drug addiction has not been effectively treated, which may be due to the difficulty in finding suitable targets to treat this complex disease, thus increasing the need for further identification of new therapeutic methods (Guo et al., 2017). Based on the features extracted from independent component analysis and sliding window correlation, this study determined the most relevant features for potential MA treatment and provided possible biomarkers to distinguish and predict the treatment response of MA-dependent patients. We hope in the future more large-sample studies could be conducted to help formulate effective drug treatment strategies and select surgical targets for MA treatment.

## Conclusion

In this study, we successfully distinguished the MA abstainers from the normal control group, and determined the abnormally changed parts of brain networks through analyzing the RS-fMRI images of the whole brain. Our results prove the effectiveness the classification algorithm. Therefore, we believe that people who have been detoxified for a period of time still have differences in the time domain in DDMN.

## Data Availability Statement

The data analyzed in this study is subject to the following licenses/restrictions: This data set is provided by Xiangya Second Hospital. Requests to access these datasets should be directed to TD, dongtingting@csu.edu.cn.

## Ethics Statement

The studies involving human participants were reviewed and approved by National Clinical Research Center for Mental Disorders, and Department of Psychiatry, The Second Xiangya Hospital of Central South University, Changsha, Hunan. The patients/participants provided their written informed consent to participate in this study. Written informed consent was obtained from the individual(s) for the publication of any potentially identifiable images or data included in this article.

## Author Contributions

TD wrote and experimented the manuscript. YT, QH, SH, JX, YG, QJ, HS, and HZ revised the manuscript and collected the data. All authors contributed to the article and approved the submitted version.

## Conflict of Interest

The authors declare that the research was conducted in the absence of any commercial or financial relationships that could be construed as a potential conflict of interest.

## Publisher’s Note

All claims expressed in this article are solely those of the authors and do not necessarily represent those of their affiliated organizations, or those of the publisher, the editors and the reviewers. Any product that may be evaluated in this article, or claim that may be made by its manufacturer, is not guaranteed or endorsed by the publisher.

## Footnotes

1
https://www.fil.ion.ucl.ac.uk/spm/software/spm12

2
http://mialab.mrn.org/software/gift/

3
http://mrvar.fdv.uni-lj.si/pajek/

## References

[B1] Abrol A. Damaraju E. Miller R. L. Stephen J. M. Claus E. D. Mayer A. R. (2017). Replicability of time-varying connectivity patterns in large resting state fMRI samples. *Neuroimage* 163 160–176. 10.1016/j.neuroimage.2017.09.020 28916181 PMC5775892

[B2] Abrol A. Fu Z. Du Y. Calhoun V. D. (2019). “Multimodal data fusion of deep learning and dynamic functional connectivity features to predict Alzheimer’s disease progression,” in *Proceedings of the 41st Annual International Conference of the IEEE Engineering in Medicine and Biology Society (EMBC)*, (Berlin: Institute of Electrical and Electronics Engineers). 10.1109/EMBC.2019.8856500 31946844

[B3] Allen E. A. Damaraju E. Plis S. M. Erhardt E. B. Eichele T. Calhoun V. D. (2014). Tracking whole-brain connectivity dynamics in the resting state. *Cereb. Cortex* 24 663–676. 10.1093/cercor/bhs352 23146964 PMC3920766

[B4] Brandman T. Malach R. Simony E. (2021). The surprising role of the default mode network in naturalistic perception. *Commun. Biol.* 4:79. 33469113 10.1038/s42003-020-01602-z PMC7815915

[B5] Calhoun V. D. Adali T. Stevens M. C. Kiehl K. A. Pekar J. J. (2005). Semi-blind ICA of fMRI: a method for utilizing hypothesis-derived time courses in a spatial ICA analysis. *Neuroimage* 25 527–538. 15784432 10.1016/j.neuroimage.2004.12.012

[B6] Chen Y. Li M. Ji Q. Su Z. Yang Z. Xu Y. (2021). Clinical study of paliperidone palmitate long-acting injection combined with electroacupuncture in the treatment of methamphetamine addicts. *Front. Pharmacol.* 12:698740. 10.3389/fphar.2021.698740 34220522 PMC8245672

[B7] Damaraju E. Allen E. A. Belger A. Ford J. M. Mcewen S. Mathalon D. H. (2014). Dynamic functional connectivity analysis reveals transient states of dysconnectivity in schizophrenia. *Neuroimage Clin.* 5 298–308. 10.1016/j.nicl.2014.07.003 25161896 PMC4141977

[B8] Douglas P. K. Harris S. Yuille A. Cohen M. S. (2011). Performance comparison of machine learning algorithms and number of independent components used in fMRI decoding of belief vs. disbelief. *Neuroimage* 56 544–553. 10.1016/j.neuroimage.2010.11.002 21073969 PMC3099263

[B9] Du Y. Fan Y. (2013). Group information guided ICA for fMRI data analysis. *Neuroimage* 69 157–197. 10.1016/j.neuroimage.2012.11.008 23194820

[B10] Du Y. Pearlson G. D. Lin D. Sui J. Chen J. Salman M. (2017). Identifying dynamic functional connectivity biomarkers using GIG-ICA: application to schizophrenia, schizoaffective disorder, and psychotic bipolar disorder. *Hum. Brain Mapp.* 38 2683–2708. 10.1002/hbm.23553 28294459 PMC5399898

[B11] Fan F. Liao X. Lei T. Zhao T. Xia M. Men W. (2021). Development of the default-mode network during childhood and adolescence: a longitudinal resting-state fMRI study. *Neuroimage* 226:117581. 10.1016/j.neuroimage.2020.117581 33221440

[B12] Fayers P. M. (2008). The scales were highly correlated: P = 0.0001. *Qual. Life Res.* 17 651–652. 10.1007/s11136-008-9351-4 18461472

[B13] Glasser M. F. Coalson T. S. Bijsterbosch J. D. Harrison S. J. Harms M. P. Anticevic A. (2018). Using temporal ICA to selectively remove global noise while preserving global signal in functional MRI data. *Neuroimage* 181 692–717. 10.1016/j.neuroimage.2018.04.076 29753843 PMC6237431

[B14] Guo H. Zhang F. Chen J. Xu Y. Xiang J. (2017). Machine learning classification combining multiple features of a hyper-network of fMRI data in Alzheimer’s disease. *Front. Neurosci.* 11:615. 10.3389/fnins.2017.00615 29209156 PMC5702364

[B15] Hehn T. M. Kooij J. F. P. Hamprecht F. A. (2019). End-to-end learning of decision trees and forests. *Int. J. Comp. Vis.* 128 997–1011. 10.1007/s11263-019-01237-6

[B16] Higgins C. Liu Y. Vidaurre D. Kurth-Nelson Z. Dolan R. Behrens T. (2021). Replay bursts in humans coincide with activation of the default mode and parietal alpha networks. *Neuron* 109:e887. 10.1016/j.neuron.2020.12.007 PMC7927915 33357412

[B17] Himberg J. Hyvarinen A. Esposito F. (2004). Validating the independent components of neuroimaging time series via clustering and visualization. *Neuroimage* 22 1214–1222. 10.1016/j.neuroimage.2004.03.027 15219593

[B18] Huang J. Zhang R. Wang S. Zhang D. Leung C. K. Yang G. (2021). Methamphetamine and HIV-tat protein synergistically induce oxidative stress and blood-brain barrier damage via transient receptor potential melastatin 2 channel. *Front. Pharmacol.* 12:619436. 10.3389/fphar.2021.619436 33815104 PMC8010131

[B19] Jiang P. Sun J. Zhou X. Lu L. Li L. Huang X. (2021). Functional connectivity abnormalities underlying mood disturbances in male abstinent methamphetamine abusers. *Hum. Brain Mapp.* 42 3366–3378. 10.1002/hbm.25439 33939234 PMC8249885

[B20] Keilholz S. D. Magnuson M. E. Pan W. J. Willis M. Thompson G. J. (2013). Dynamic properties of functional connectivity in the rodent. *Brain Connect.* 3 31–40. 10.1089/brain.2012.0115 23106103 PMC3621313

[B21] Li H. Chen J. A. Ding Q. Z. Lu G. Y. Wu N. Su R. B. (2021). Behavioral sensitization induced by methamphetamine causes differential alterations in gene expression and histone acetylation of the prefrontal cortex in rats. *BMC Neurosci.* 22:24. 10.21203/rs.2.20165/v3 33823794 PMC8022387

[B22] Li Y. O. Adali T. Calhoun V. D. (2007). Estimating the number of independent components for functional magnetic resonance imaging data. *Hum. Brain Mapp.* 28 1251–1266. 10.1002/hbm.20359 17274023 PMC6871474

[B23] Liu Y. Zhu J. Li Q. Wang Y. Li Y. Chen J. (2020). Differences in the amplitude of low-frequency fluctuation between methamphetamine and heroin use disorder individuals: a resting-state fMRI study. *Brain Behav.* 10:e01703. 32666687 10.1002/brb3.1703 PMC7507466

[B24] Luo N. Tian L. Calhoun V. D. Chen J. Lin D. Vergara V. M. (2019). Brain function, structure and genomic data are linked but show different sensitivity to duration of illness and disease stage in schizophrenia. *Neuroimage Clin.* 23:101887. 10.1016/j.nicl.2019.101887 31176952 PMC6558215

[B25] Maniar Y. M. Peck K. K. Jenabi M. Gene M. Holodny A. I. (2021). Functional MRI shows altered deactivation and a corresponding decrease in functional connectivity of the default mode network in patients with gliomas. *AJNR Am. J. Neuroradiol.* 42 1505–1512. 10.3174/ajnr.A7138 33985945 PMC8367628

[B26] Manzanares J. Cabanero D. Puente N. Garcia-Gutierrez M. S. Grandes P. Maldonado R. (2018). Role of the endocannabinoid system in drug addiction. *Biochem. Pharmacol.* 157 108–121. 10.1016/j.bcp.2018.09.013 30217570

[B27] Nicolas C. Hofford R. S. Dugast E. Lardeux V. Belujon P. Solinas M. (2021). Prevention of relapse to methamphetamine self-administration by environmental enrichment: involvement of glucocorticoid receptors. *Psychopharmacology.* Mar 25. 10.1007/s00213-021-05770-6 33768375

[B28] Peng Y. Zhang S. Zhou Y. Song Y. Yang G. Hao K. (2021). Abnormal functional connectivity based on nodes of the default mode network in first-episode drug-naive early-onset schizophrenia. *Psychiatry Res.* 295:113578. 10.1016/j.psychres.2020.113578 33243520

[B29] Plis S. M. Amin M. F. Chekroud A. Hjelm D. Damaraju E. Lee H. J. (2018). Reading the (functional) writing on the (structural) wall: multimodal fusion of brain structure and function via a deep neural network based translation approach reveals novel impairments in schizophrenia. *Neuroimage* 181 734–747. 10.1016/j.neuroimage.2018.07.047 30055372 PMC6321628

[B30] Qiu Y. Lin Q. H. Kuang L. D. Gong X. F. Cong F. Wang Y. P. (2019). Spatial source phase: a new feature for identifying spatial differences based on complex-valued resting-state fMRI data. *Hum. Brain Mapp.* 40 2662–2676. 10.1002/hbm.24551 30811773 PMC6865858

[B31] Rashid B. Arbabshirani M. R. Damaraju E. Cetin M. S. Miller R. Pearlson G. D. (2016). Classification of schizophrenia and bipolar patients using static and dynamic resting-state fMRI brain connectivity. *Neuroimage* 134 645–657. 10.1016/j.neuroimage.2016.04.051 27118088 PMC4912868

[B32] Salimi-Khorshidi G. Douaud G. Beckmann C. F. Glasser M. F. Griffanti L. Smith S. M. (2014). Automatic denoising of functional MRI data: combining independent component analysis and hierarchical fusion of classifiers. *Neuroimage* 90 449–468. 10.1016/j.neuroimage.2013.11.046 24389422 PMC4019210

[B33] Salman M. S. Du Y. Lin D. Fu Z. Damaraju E. Sui J. (2018). Group ICA for identifying biomarkers in schizophrenia: ‘Adaptive’ networks via spatially constrained ICA show more sensitivity to group differences than spatio-temporal regression. *NeuroImage Clin.* 22:101747. 10.1016/j.nicl.2019.101747 30921608 PMC6438914

[B34] Shakil S. Lee C. H. Keilholz S. D. (2016). Evaluation of sliding window correlation performance for characterizing dynamic functional connectivity and brain states. *Neuroimage* 133 111–128. 10.1016/j.neuroimage.2016.02.074 26952197 PMC4889509

[B35] Shakil S. Magnuson M. E. Keilholz S. D. Lee C.-H. (2014). Cluster-based analysis for characterizing dynamic functional connectivity. *Annu. Int. Conf. IEEE Eng. Med. Biol. Soc.* 2014 982–985. 25570125 10.1109/EMBC.2014.6943757 PMC4457444

[B36] Stoehr C. Aston J. A. D. Kirch C. (2021). Detecting changes in the covariance structure of functional time series with application to fMRI data. *Econom. Stat.* 18 44–62. 10.1016/j.ecosta.2020.04.004

[B37] Su H. Liu Y. Yin D. Chen T. Li X. Zhong N. (2020). Neuroplastic changes in resting-state functional connectivity after rTMS intervention for methamphetamine craving. *Neuropharmacology* 175:108177. 10.1016/j.neuropharm.2020.108177 32505485

[B38] Taheri S. Xun Z. See R. E. Joseph J. E. Reichel C. M. (2016). Cocaine and methamphetamine induce opposing changes in BOLD signal response in rats. *Brain Res.* 1642 497–504. 10.1016/j.brainres.2016.04.040 27103569 PMC4899179

[B39] Tang Y. Liu B. Yang Y. Wang C. M. Meng L. Tang B. S. (2018). Identifying mild-moderate Parkinson’s disease using whole-brain functional connectivity. *Clin. Neurophysiol.* 129 2507–2516. 10.1016/j.clinph.2018.09.006 30347309

[B40] Thompson G. J. Magnuson M. E. Merritt M. D. Schwarb H. Pan W. J. Mckinley A. (2013). Short-time windows of correlation between large-scale functional brain networks predict vigilance intraindividually and interindividually. *Hum. Brain Mapp.* 34 3280–3298. 10.1002/hbm.22140 22736565 PMC6870033

[B41] Vakamudi K. Trapp C. Talaat K. Gao K. Sa De La Rocque Guimaraes B. Posse S. (2020). Real-time resting-state functional magnetic resonance imaging using averaged sliding windows with partial correlations and regression of confounding signals. *Brain Connect.* 10 448–463. 10.1089/brain.2020.0758 32892629 PMC7580636

[B42] van Buuren M. Lee N. C. Vegting I. Walsh R. J. Sijtsma H. Hollarek M. (2021). Intrinsic network interactions explain individual differences in mentalizing ability in adolescents. *Neuropsychologia* 151:107737. 10.1016/j.neuropsychologia.2020.107737 33383039

[B43] Wilson R. S. Mayhew S. D. Rollings D. T. Goldstone A. Przezdzik I. Arvanitis T. N. (2015). Influence of epoch length on measurement of dynamic functional connectivity in wakefulness and behavioural validation in sleep. *Neuroimage* 112 169–179. 10.1016/j.neuroimage.2015.02.061 25765256

[B44] Xie J. Douglas P. K. Wu Y. N. Brody A. L. Anderson A. E. (2017). Decoding the encoding of functional brain networks: an fMRI classification comparison of non-negative matrix factorization (NMF), independent component analysis (ICA), and sparse coding algorithms. *J. Neurosci. Methods* 282 81–94. 10.1016/j.jneumeth.2017.03.008 28322859 PMC5507942

[B45] Yan C. G. Wang X. D. Zuo X. N. Zang Y. F. (2016). DPABI: data processing & analysis for (resting-state) brain imaging. *Neuroinformatics* 14 339–351. 10.1007/s12021-016-9299-4 27075850

[B46] Yan W. Calhoun V. Song M. Cui Y. Yan H. Liu S. (2019). Discriminating schizophrenia using recurrent neural network applied on time courses of multi-site FMRI data. *Ebiomedicine* 47 543–552. 10.1016/j.ebiom.2019.08.023 31420302 PMC6796503

[B47] Yuan Y. Pan X. Wang R. (2021). Biophysical mechanism of the interaction between default mode network and working memory network. *Cogn. Neurodyn.* 10.1007/s11571-021-09674-1 PMC8572310 34786031

[B48] Zalesky A. Breakspear M. (2015). Towards a statistical test for functional connectivity dynamics. *Neuroimage* 114 466–470. 10.1016/j.neuroimage.2015.03.047 25818688

[B49] Zhang W. Lv J. Li X. Zhu D. Jiang X. Zhang S. (2019). Experimental comparisons of sparse dictionary learning and independent component analysis for brain network inference from fMRI data. *IEEE Trans. Biomed. Eng.* 66 289–299. 10.1109/tbme.2018.2831186 29993466

